# Entropy, Fluctuations, and Disordered Proteins

**DOI:** 10.3390/e21080764

**Published:** 2019-08-06

**Authors:** Eshel Faraggi, A. Keith Dunker, Robert L. Jernigan, Andrzej Kloczkowski

**Affiliations:** 1 Department of Physics, Indiana University Purdue University Indianapolis, Indianapolis, IN 46202, USA; 2 Research and Information Systems, LLC, 1620 E. 72nd ST., Indianapolis, IN 46240, USA; 3 Department of Biochemistry and Molecular Biology, Indiana University School of Medicine, Indianapolis, IN 46202, USA; 4 Roy J. Carver Department of Biochemistry, Biophysics and Molecular Biology, Iowa State University, Ames, IA 50011, USA; 5 Battelle Center for Mathematical Medicine, The Research Institute at Nationwide Children’s Hospital, Columbus, OH 43205, USA; 6 Department of Pediatrics, The Ohio State University, Columbus, OH 43205, USA

**Keywords:** protein disorder, protein structure, entropy, fluctuations, mutations

## Abstract

Entropy should directly reflect the extent of disorder in proteins. By clustering structurally related proteins and studying the multiple-sequence-alignment of the sequences of these clusters, we were able to link between sequence, structure, and disorder information. We introduced several parameters as measures of fluctuations at a given MSA site and used these as representative of the sequence and structure entropy at that site. In general, we found a tendency for negative correlations between disorder and structure, and significant positive correlations between disorder and the fluctuations in the system. We also found evidence for residue-type conservation for those residues proximate to potentially disordered sites. Mutation at the disorder site itself appear to be allowed. In addition, we found positive correlation for disorder and accessible surface area, validating that disordered residues occur in exposed regions of proteins. Finally, we also found that fluctuations in the dihedral angles at the original mutated residue and disorder are positively correlated while dihedral angle fluctuations in spatially proximal residues are negatively correlated with disorder. Our results seem to indicate permissible variability in the disordered site, but greater rigidity in the parts of the protein with which the disordered site interacts. This is another indication that disordered residues are involved in protein function.

## 1. Introduction

Protein disorder, where whole proteins or protein segments are either unstable or meta-stable, has proven to be a critical property to understand function in biological systems [1,2,3,4,5,6,7,8,9,10,11,12,13,14,15,16,17,18,19,20,21,22,23,24,25,26,27,28,29,30,31,32,33]. Such disordered proteins and regions have been demonstrated to become more abundant as organism complexity increases [23,29,34,35,36,37]. This increase in disorder with organism complexity likely results from the key roles played by disorder in the signaling and regulatory processes underlying cellular differentiation, cell cycle control, gene regulation, and protein–protein interactions, especially enabling the existence of hubs [10,13,38,39,40,41,42,43,44]. The origin of these effects arises because disordered proteins enable more diverse function, yet are still able to maintain a high degree of specialization in their specific interactions.

Understanding entropic effects in protein systems is usually difficult, and understanding molecular stability is arguably one of the most important problems in molecular biology, particularly of interest for proteins. This problem is clearly important for understanding the relationship between protein function, structure, and sequence. The full knowledge of protein stabilities requires the reliable evaluation of energies and entropies. This would also aid in the evaluation of structural models of the large number of genes with unknown protein structures. Even a partial understanding of the relationship between entropic sequence effects and structure has already yielded significant success [45,46,47], and will most likely lead to further success in the future.

Entropy and disorder are intimately related. The aim here was to explore this relationship for proteins. In this work, we analyzed datasets of related proteins with known sequences and structures. We split each cluster of related proteins into two sets, one having a greater sequence similarity than the other. We do this as a way to further explore the relationship to sequence variability. We analyzed the variations in sequence and structure among these sets and quantify their entropies.

Entropy is a global variable. It is the logarithm of the number of phase space states accessible to a system. In very large systems, such that they appear continuous, the number of states is estimated from the phase space volume. Since velocity coordinates in a protein system are related to thermal degrees of freedom and we assumed a constant temperature, the momentum distribution of different conformational states will be similar and, to a good approximation, the difference in the entropy of two states will depend only on the difference between their configuration space volumes.

A previous study of entropy in protein systems by Franzosa and Xia [48] investigated the constraints that structure imposes upon protein evolution. They found that solvent exposure is the most significant structural determinant of residue evolution and also identified a weak effect from the packing density. The relationship between solvent exposure and entropy they found was “strong, positive, and linear”. We investigated these relationships.

## 2. Materials and Methods

To obtain a dataset of both sequence and structure of related proteins, we clustered PDB [49,50,51] structures with resolution better than 3 Angstrom, at 25% or greater sequence identity (SID). This was done by first clustering the PDB at 99% SID, to remove redundancies, and then clustering at 25% SID. We used BLASTClust [52] with default parameters to do the clustering. We selected the largest clusters and divided each cluster into two separate sets having SID values in the range of 30–50% SID for one set (A types) and 60–80% for the second set (B types). These collections represent more diverse A sets and less diverse B sets, respectively.

**Figure 1:**
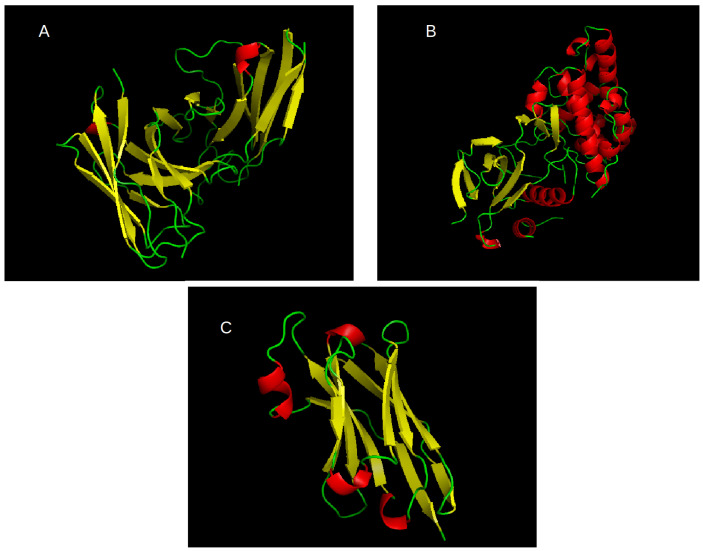
Cartoons of the structures for the seeds for the sets L1, L2, and L3. (**A**) Seed for the L1 sets L1A and L1B, an antibody fragment, PDBID: 5U68E. (**B**) Seed for the L2 sets L2A and L2B, JNK3 mitogen-activated protein kinase 10, PDBID: 3TTIA. (**C**) Seed for the L3 sets L3A and L3B, nanobody MU551, PDBID: 5F1OB. The color scheme is according to the secondary structure types, with beta strands yellow, helix red and coil green. Note that we keep a dark background to aid in viewing loops and especially loops with missing residues.

The most abundant cluster of related proteins we found is that of antibodies. We use the notation of L1 to refer to this set. The PDB ID for the structural seed of this set is 5U68E, where the fifth character gives the chain ID. The second most abundant cluster is for kinases. We use the notation of L2 to refer to this set. The PDB ID for the structural seed of this set is 3TTIA. Per review request, we included the third largest cluster as an additional test for some of our results as described in the text. The PDB seed for this cluster is 5F1OB and it is labeled L3. Cartoon representations of the seed structures for L1, L2, and L3, are given in Figure 1. In total, we have 6 sets of proteins: L1, L2, and L3, each split into two sets as described above. The number of proteins in each set and the corresponding descriptors are given in Table 1.

For each of these sets, we collected their sequences and executed a multiple sequence alignment (MSA) using CLUSTALW [53,54] with default parameters. The length of the alignment obtained for each set is also given in Table 1. We observed that, the more varied is the set, the longer is the length of the alignment, as expected. In addition, we found that the more distant are the proteins (lower SID), the longer is the alignment. That is also to be expected since aligning more distant proteins would require inserting more gaps to accommodate the larger sequence variations. We refer to a given column in the MSA as an MSA site. To avoid cases of sparseness in the data, we only used MSA sites having a count of at least 20 amino acids. The number of MSA sites obeying this condition is also given in Table 1. Finally, we give the means, medians, and standard deviations, for the TM-scores [55] between the seed structure and all the rest of the structures in the corresponding set. The Template Modeling score (TM-score) is a parameter to measure protein structure similarity. It is calculated from the distances between the residues of two aligned proteins:

(1) $$TM-score=max[\frac{1}{L_{t}}\sum_{i=1}^{L_{a}}\frac{1}{1+{\left(\frac{d_{i}}{d_{0}}\right)}^{2}}],$$

where the maximum is taken over all possible alignments. $L_{t}$ is the length of the target protein, $L_{a}$ is the length of the protein that is aligned to it, $d_{i}$ is the distance between the residues at alignment location *i*, and $d_{0}$ is a scaling distance, optimized to $d_{0}=1.24\sqrt[{3}]{L_{t}-15}-1.8$. With such calibration, the TM-score does not depend on the protein length, and varies between 0 and 1, with 1 indicating a perfect match. A TM-score below 0.2 indicates structurally unrelated proteins, while a TM-score greater than 0.5 indicates the two proteins belong to the same fold.

We observed that the largest set L1 is distributed around relatively distinct structures with some similarity, having a TM-score of just below 0.5. All three sets have TM-scores with means ranging between 0.37 and 0.67. We also observed an appropriate increase in mean and median TM-score for the more similar structures (the B sets - SID 60–80%). We note that the set L2B with SID 60–80% is composed of two overlapping structure clusters. This is exhibited in the range of TM-score and also somewhat skews the results for this set.

To evaluate the amount of disorder at a given MSA site, we introduce the parameter δ that counts the excess number of disordered residues:

(2) $$\delta =\frac{N_{dis}-N_{ord}}{N_{res}}.$$

Here, $N_{dis}$ is the number of residues at an MSA site classified as disordered, i.e., their coordinates appear in the missing coordinates section of the corresponding PDB file (remark 465), and $N_{ord}$ is the number of residues classified as ordered, i.e., with coordinates in the corresponding PDB file. $N_{res}$ is the total number of residues per MSA site, i.e., the total number of sequences that have a residue at this site in the MSA. Note that in a few instances there is a discrepancy between the residue type as it appears in the Uniprot [56] sequence information and that in the PDB file. We discarded these cases and did not classify them as either ordered or disordered, however, they appear in $N_{res}$. Hence, in general $N_{res}\geq N_{dis}+N_{ord}$, and −1≤δ≤1. At a given MSA site, δ=1 indicates all residues at that site are classified as disordered, while δ=−1 indicates that all residues at that site are classified as ordered. Using this approach, for any specific mutation site, we established linked measures of both disorder and structural order. Note that, for a given MSA site, with a number of proteins having a residue at that location, those residues with missing coordinates in the PDB file are labeled disordered. For those residues with coordinates in the PDB, we calculated the structural features. Hence, for a given MSA site, we have both structural and disorder information and we investigated the relationship between them. To ensure that we have some sampling points per MSA site we restricted our attention only to those MSA sites that have at least 20 proteins contributing a residue to the alignment. Note that, since the protein sequences were obtained from Uniprot, residues without PDB coordinates can still contribute to the alignment.

To characterize the structure and fluctuations at a given MSA site, we proceeded as follows. We started by finding the closest long-range contact (CLRC), that is, the closest residue in space which has a sequence separation of more than 5 residues from the MSA site. The distances between the C${}^{\beta }$ (C${}^{\alpha }$ for GLY) atoms was used to measure the distance between residues. For all MSA sites, we calculated the average of these distances, $d_{av}$, and their standard deviations, $d_{sd}$. We quantified the rotational relationship by calculating the cosines of the angles between the N-C${}^{\alpha }$, C${}^{\alpha }$-C, and C${}^{\alpha }$-C${}^{\beta }$ (0.0 for GLY) bonds for the residue pair identified by a given MSA site and its CLRC site. The cosine of the angle was obtained by taking the dot product of the bond vectors and normalizing by their lengths. The average values of these were identified as $c1_{av}$, $c2_{av}$, $c3_{av}$, respectively, and the standard deviations of the cosines of these angles are denoted as $c1_{sd}$, $c2_{sd}$, and $c3_{sd}$, respectively.

**Figure 2:**
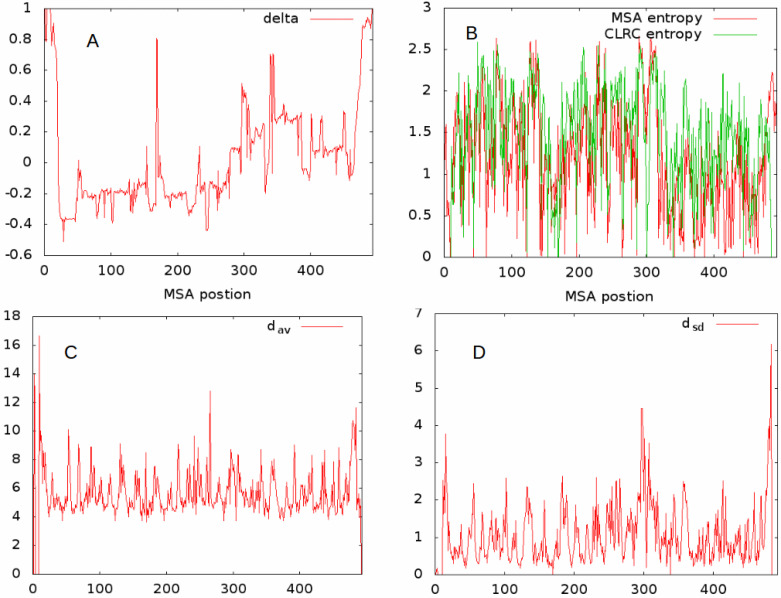
Example plots of: δ (**A**); the two entropies $s_{1}$ and $s_{2}$ (**B**); $d_{av}$ (**C**); and $d_{sd}$ (**D**), for all MSA sites having at least 20 residues contributing to the alignment for the set L1A.

We also calculated the Shannon entropy for the original MSA site with respect to residue type fluctuations, i.e., from those aligned sequences at a given MSA site we created a probability distribution for residue type and then used ∑pln(p) to calculate the sequence entropy. We performed a similar procedure on the probability distribution for residue types of the CLRC. The Shannon entropy parameter for the original MSA site is denoted $s_{1}$, and that for the CLRC $s_{2}$. In Figure 2, we give example plots of δ (Figure 2A), the two entropies $s_{1}$ and $s_{2}$ (Figure 2B), $d_{av}$ (Figure 2C), and $d_{sd}$ (Figure 2D), for all MSA sites having at least 20 residues contributing to the alignment for the set L1A. The relationships even between this limited set seem complex. In what follows, we try to further characterize the features of a given MSA site, and as a first approach determine the correlations between these different features.

In addition, we calculated the average and standard-deviations for the accessible surface area, relative accessible surface area (RSA), and the ϕ and ψ dihedral angles for both the MSA site and the CLRC site. For the MSA site, we identify these parameters as $a1_{av}$, $a1_{sd}$, $ra1_{av}$, $ra1_{sd}$, $\phi 1_{av}$, $\phi 1_{sd}$, $\psi 1_{av}$, and $\psi 1_{sd}$, respectively. For the CLRC, we identify these as $a2_{av}$, $a2_{sd}$, $ra2_{av}$, $ra2_{sd}$, $\phi 2_{av}$, $\phi 2_{sd}$, $\psi 2_{av}$, and $\psi 2_{sd}$, respectively. We also calculated the propensity of secondary structure types at the MSA and CLRC sites. In general, we use the index 1 for the MSA site and the index 2 for the CLRC site. The letters *h, c, and e* refer to the helix, coil, and, sheet secondary structure types, respectively. We use the same secondary structure assignment scheme as in SPINE-X [57,58,59]. From these propensities, we calculate a probability distribution for secondary structure states and from that we calculate the Shannon entropy for secondary structure. We use the notation $s_{ss1}$ for this entropy for the MSA site and $s_{ss2}$ for this entropy at the CLRC.

## 3. Results and Discussion

To estimate the relationships between the various parameters and the disorder propensity of an alignment site, δ, we calculated Pearson correlation coefficients (correlations) between them. In Table 2, Table 3, Table 4, Table 5, Table 6 and Table 7, we give the correlations between the various sequence and structure parameters we calculated. Correlations to entropic parameters are given in Table 2. We found that there is a consistent negative correlation between $s_{2}$ and δ. The only exception is a marginal positive correlation for L3B. Since L3B is a smaller set, this result may be due to not enough statistics. The positive correlation between $s_{2}$ and δ seems to indicate a significant average residue conservation for residues proximate to disordered sites. This can be an indication of their importance for function, and this will be further explored in future work. The correlation values between δ and the entropy of the disordered site are weaker. This indicates that the residue type substitution rate is not significantly different between ordered and disordered residues in this case. This behavior of the correlation could come about because disordered sites form structure with protein or nucleic acid partners, with the resulting structure imparting increased conservation for the amino acids involved in the formation of the complex. Some disordered regions have high conservation throughout [60], possibly because they form multiple partnerships such that nearly all of their residues are important for at least one critical structure.

In Table 3, we give the correlations between the different entropic parameters. Overall, there is a positive correlation between them. In addition, as expected, correlations within the more similar set L2B appear larger than those of the less similar sets L2A. For set L1, the situation is less clear and may be an indication that set L1 carries more noise.

Correlations between δ and spatial parameters are given in Table 4. We found significant positive correlations between δ and $d_{sd}$. This is to be expected from the definition of disorder. It is interesting to note that the correlations to the fluctuations of the rotational degrees of freedom ($c1_{sd}$, $c2_{sd}$, $c3_{sd}$) are all negative. This is a puzzle since it seems to associate larger rotational fluctuations with less disorder. It may be an indication that disorder fluctuations are more abundant in the radial directions than in the rotational ones.

To further test the correlation between δ and $d_{av}$ and $d_{sd}$, we performed a similar analysis on the third most abundant cluster of related sequences of structures deposited in the PDB. The general properties for this cluster are labeled under L3 in Table 1. The seed PDB structure for this protein chain is 5F1OB; a representative cartoon of it is given in Figure 1. For the more diverse subgroup of L3 (30–50% SID), we found correlations of 0.219 and 0.195 for $d_{av}$ and $d_{sd}$, respectively. For the less diverse subgroup of L3 (60–80% SID), we found correlations of 0.361 and 0.225 for $d_{av}$ and $d_{sd}$, respectively. These trends are in line with the results for clusters L1 and L2. To estimate the statistical significance of the observed positive correlations between δ and $d_{av}$ and $d_{sd}$, we performed the following analysis for the set L1A. We started by selecting a random subset of 200 points and calculated the correlation for this subset. We then repeated this process 15 times and use a majority vote to determine the sign of the correlation. Hence, we can consider such a round a flip of a coin, with two possible outcomes. We conducted 10 such rounds and obtained a positive correlation for all of them. In analogy with coins, this would correspond to a *p*-value of $2^{-10}$, indicating confident rejection of the null hypothesis that the correlations are random.

Correlations between δ and accessible surface area parameters are given in Table 5. They are mostly significantly positive, and similarly for the RSA. This is in agreement with the general observation that disordered residues occur in exposed regions of proteins. The negative correlations with fluctuations in accessible surface area may be due to the same observation, as disordered residues would tend to remain exposed, and hence have reduced fluctuations. We could not identify any consistent trend from the correlations with the dihedral angles values (Table 6). However, we did find one for the fluctuations in the dihedral angles. Fluctuations of the dihedral angles are positively correlated with disorder propensity for the original residues, and negatively correlated for the CLRC residues. This is in agreement with the results for the Shannon entropy at these sites, indicating allowed variability at the disordered site, and increased rigidity in the parts of the protein where this disordered site interacts. This may be another indication that disordered residues are involved in protein function.

Correlations between δ and probabilities of secondary structure types are given in Table 7. As expected, we found a significant negative correlation. We found the strongest negative correlation with the propensity of β-sheets. This is also expected. We also found a negative correlation between the entropy of secondary structure and δ for both the original site and its CLRC. This indicates that as disorder is increased at a given MSA site, it becomes more probable for the secondary structure to be of a particular type. This results is consistent with our previous observations for the CLRC. It is difficult to evaluate their significance for the original site since, we have a mixture of disorder and order information.

**Figure 3:**
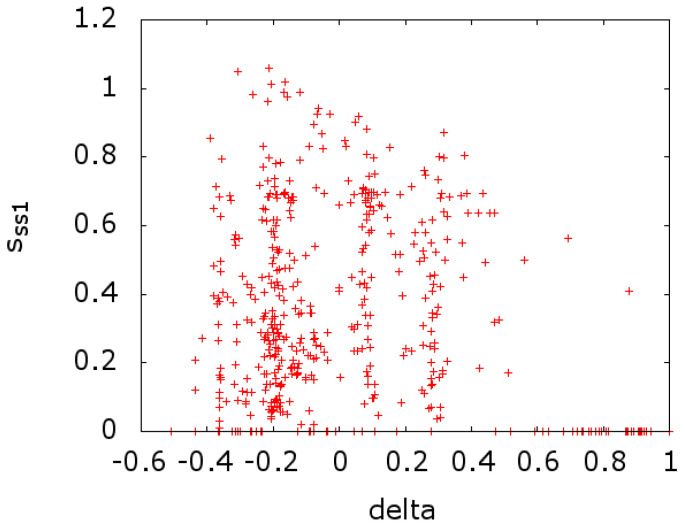
The entropy of secondary structure at a given MSA site versus the value of δ at that site.

**Figure 4:**
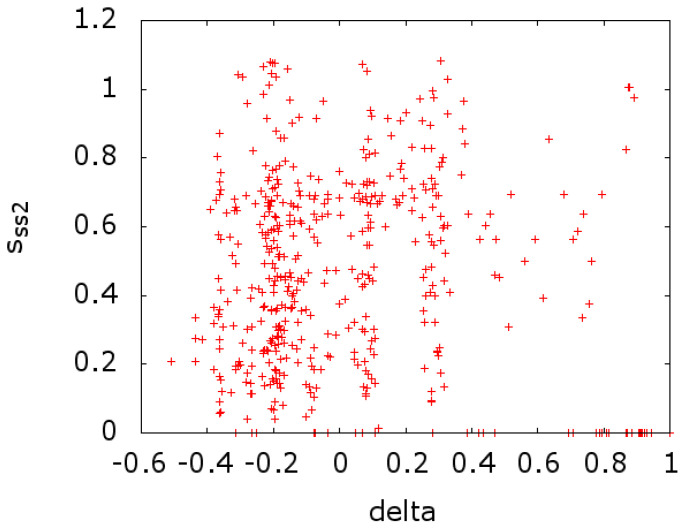
The entropy of secondary structure at the CLRC to the MSA site versus the value of δ at that site.

In Figure 3, we plot the entropy of secondary structure at a given MSA site ($s_{ss1}$) versus the value of δ at that site. In Figure 4, we plot the same at the CLRC site ($s_{ss2}$ versus δ). In both cases, we see a scatter of points on the *x*-axis. This indicates a strong effect that is due to conserved sites with zero entropy. If we remove these sites from the calculations of the entropy, we get a reversal in the sign of the correlation, going from −0.213 and −0.141 to 0.139 and 0.247 for Figure 3 and Figure 4, respectively.

We also calculated the correlations between secondary structure type probabilities of the original site and the CLRC. Our aim here was to study the relationship between the structure at the MSA and CLRC sites. Specifically, if there is a difference in that relationship between sites that tend to be more or less disordered. In Table 8, we give these values. We also calculated these correlation values separately for MSA sites with δ≥0 (more disordered) and with δ<0 (more ordered). There is a clear positive correlation for secondary structure types regardless of the state of disorder. One should note that for set L1 there is very little helix conformation, as observed in Figure 1A. This is the reason for the low correlation for this case in Table 8 as there are not enough data.

Finally, part of our aim in the research was to find differences in behavior between two sets of proteins, one with more related proteins, and the other a more diverse set of proteins. Unfortunately, we do not feel confident in drawing conclusions from the data regarding that question. However, future studies may find the data presented here useful.

## 4. Conclusions

We investigated the relationship between entropy and disorder using native protein structures found in the PDB. By finding clusters of related proteins and studying the MSA of the sequences of these clusters, we were able to establish a link between sequence, structure, and disorder information. We introduced several parameters as measures of fluctuations at a given MSA site and used these as plausible representative of the sequence and structure entropy at that site. We then defined a disorder propensity of an MSA site, δ, and calculated the Pearson correlations between it and our fluctuation parameters. Overall, we found a tendency for negative correlations between disorder and structure. We also found evidence for residue-type conservation for those residues in close proximity to potentially disordered sites. Mutations at the disordered site itself appear to be allowed.

We found significant positive correlations between δ and the fluctuations in the system. This is to be expected from the definition of disorder. It is interesting to note that the correlations to the fluctuations of the rotational degrees of freedom ($c1_{sd}$, $c2_{sd}$, and $c3_{sd}$) are all negative. This may be an indication that disorder fluctuations are more abundant in the radial direction than in rotational directions but this result will be investigated in future studies.

As expected, we found positive correlations for disorder and accessible surface area, indicating that disordered residues occur in exposed regions of proteins. We found a negative correlations for disorder with fluctuations in accessible surface area. This seems to indicate that disordered residues would tend to remain exposed, and hence with reduced RSA fluctuations. We also found that fluctuations in the dihedral angles at the original mutated residue and disorder are positively correlated while dihedral angle fluctuations in the CLRC residue are negatively correlated with disorder. This agrees with the results for the Shannon entropy at these sites, indicating permissible variability in the disordered site, but greater rigidity in the parts of the protein with which the disordered site interacts. This is another indication that disordered residues are involved in protein function. We also found indications that, as disorder is increased at a given MSA site, it becomes more probable for the secondary structure to be of a particular type.

## Figures and Tables

**Table 1:** Properties of the protein sets used.

Set:	L1		L2		L3	
SID to seed:	30–50%	60–80%	30–50%	60–80%	30–50%	60–80%
Number of proteins	1759	586	398	261	378	393
Length of MSA	666	565	930	498	535	228
>20 MSA sites	494	397	569	157	355	182
TMS mean	0.45	0.49	0.51	0.37	0.53	0.67
TMS median	0.43	0.49	0.24	0.36	0.23	0.87
TMS STDEV	0.06	0.06	0.26	0.05	0.32	0.30
Number of residues in seed protein	294		464		163	
Seed PDBID	5U68E		3TTIA		5F1OB	
Function title	Antibody fragment		JNK3 mitogen-activated kinase		Nanobody MU551	

Properties of the most abundant clusters of related proteins in the PDB.

**Table 2:** Correlations between the disorder propensity δ and entropic parameters.

Parameter	L1		L2		L3	
	30–50%	60–80%	30–50%	60–80%	30–50%	60–80%
$s_{1}$	−0.074	0.092	0.030	0.240	0.020	0.250
$s_{2}$	−0.402	−0.316	−0.346	−0.286	−0.145	0.042
$s_{ss1}$	−0.213	−0.138	0.050	−0.077	−0.205	0.080
$s_{ss2}$	−0.141	−0.216	−0.039	−0.101	−0.128	0.022

$s_{1}$ and $s_{2}$ are entropies with respect to fluctuations in residue type for the MSA and CLRC sites, respectively, and $s_{ss1}$ and $s_{ss2}$ are entropies with respect to fluctuations in secondary structure assignment for the MSA and CLRC sites, respectively.

**Table 3:** Correlations between the entropic parameters.

Parameter	L1		L2	
	30–50%	60–80%	30–50%	60–80%
$s_{1},s_{2}$	0.467	0.319	0.526	0.681
$s_{1},s_{ss1}$	0.267	0.179	0.280	0.403
$s_{1},s_{ss2}$	0.207	0.206	0.280	0.544
$s_{2},s_{ss1}$	0.442	0.424	0.284	0.483
$s_{2},s_{ss2}$	0.562	0.601	0.458	0.745
$s_{ss1},s_{ss2}$	0.375	0.370	0.374	0.549

Correlation between the different entropic parameters calculated for the different sets of aligned proteins.

**Table 4:** Correlations between the disorder propensity δ and spatial parameters.

Parameter	L1		L2	
	30–50%	60–80%	30–50%	60–80%
$d_{av}$	0.082	−0.097	0.354	0.296
$d_{sd}$	0.191	0.114	0.425	0.248
$c1_{av}$	0.259	0.181	0.026	0.147
$c1_{sd}$	−0.131	−0.163	−0.013	−0.182
$c2_{av}$	0.247	0.142	0.052	0.236
$c2_{sd}$	−0.137	−0.148	−0.042	−0.143
$c3_{av}$	−0.050	−0.097	0.061	−0.237
$c3_{sd}$	−0.243	−0.159	−0.002	−0.161

Correlations between spatial characteristics and the disorder propensity at a given MSA site.

**Table 5:** Correlations between the disorder propensity δ and ASA parameters.

Parameter	L1		L2	
	30–50%	60–80%	30–50%	60–80%
$a1_{av}$	0.167	0.093	0.388	0.450
$a1_{sd}$	−0.077	−0.051	0.340	0.120
$a2_{av}$	0.129	−0.040	0.317	0.253
$a2_{sd}$	−0.153	−0.235	0.215	−0.078
$ra1_{av}$	0.180	0.090	0.409	0.409
$ra1_{sd}$	−0.040	−0.042	0.393	0.133
$ra2_{av}$	0.163	0.006	0.320	0.214
$ra2_{sd}$	−0.163	−0.206	0.214	−0.186

Correlations between ASA characteristics and the disorder propensity at a given MSA site.

**Table 6:** Correlations between the disorder propensity δ and dihedral angle parameters.

Parameter	L1		L2	
	30–50%	60–80%	30–50%	60–80%
$\phi 1_{av}$	0.269	0.216	0.410	0.326
$\phi 1_{sd}$	0.106	0.029	0.480	−0.026
$\psi 1_{av}$	−0.006	−0.041	0.213	0.054
$\psi 1_{sd}$	0.035	0.028	0.262	0.235
$\phi 2_{av}$	0.184	0.272	−0.003	0.269
$\phi 2_{sd}$	−0.204	−0.173	−0.015	−0.314
$\psi 2_{av}$	−0.264	−0.243	−0.079	−0.292
$\psi 2_{sd}$	−0.156	−0.125	−0.082	−0.192

Correlations between dihedral angles characteristics and the disorder propensity at a given MSA site.

**Table 7:** Correlations between the disorder propensity δ and secondary structure probabilities.

Parameter	L1		L2	
	30–50%	60–80%	30–50%	60–80%
ss1h	−0.093	−0.067	−0.378	−0.120
ss1c	−0.333	−0.183	−0.303	−0.354
ss1e	−0.339	−0.189	−0.207	−0.393
ss2h	−0.104	−0.084	−0.385	−0.150
ss2c	−0.316	−0.144	−0.400	−0.319
ss2e	−0.424	−0.264	−0.294	−0.575

Correlations between secondary structure probabilities and the disorder propensity at a given MSA site.

**Table 8:** Correlations between secondary structure type probabilities.

Type	L1		L2	
	30–50%	60–80%	30–50%	60–80%
Helix	0.198	0.179	0.509	0.289
Coil	0.611	0.480	0.414	0.442
Sheet	0.741	0.716	0.643	0.620
Helix (δ≥0)	−0.033	−0.007	0.551	0.175
Coil (δ≥0)	0.566	0.332	0.893	0.993
Sheet (δ≥0)	0.768	0.585	0.721	0.658
Helix (δ<0)	0.277	0.307	0.450	0.278
Coil (δ<0)	0.588	0.559	0.341	0.354
Sheet (δ<0)	0.707	0.770	0.632	0.523

Correlations between the secondary structure probabilities of the original MSA site and its CLRC.

## Abbreviations

The following abbreviations are used in this manuscript:

SID	Sequence Identity
MSA	Multiple Sequence Alignment
CLRC	Closest Long-Range Contact
RSA	Relative Accessible Surface Area
